# Supplementary material for: In-situ probing of the Fischer-Tropsch reaction on Co single crystal surfaces up to 1 bar
Source: Nat Commun. 2025 Jan 24;16:1005. doi: 10.1038/s41467-025-56082-8 (PMC11761050; doi:10.1038/s41467-025-56082-8)
Supplement: Supplementary file 1 — Supplementary Information [file 41467_2025_56082_MOESM1_ESM.pdf]

Supplementary Information

In-situ Probing of the Fischer-Tropsch Reaction on Co Single Crystal Surfaces up to 1 bar

Lömker et al.

S1 Compiled SXRD Data

In this section we present the compiled SXRD data of the Co(0001) crystal, which was omitted from the main text due to space restrictions (See Supplementary Figure 1). For the discussion and analysis, we use the bulk hexagonal coordinate system of cobalt, with a=b=2.505 A, c = 4.089 A $\alpha = \beta$=90°, γ =120°. The reciprocal lattice coordinates are given in units of a*=b*= 2.896 A^{-1} and c*=1.537 A^{-1} ($\alpha^* = \beta^*$ =90°, γ^*=60°). The fits were performed with the ANAROD package[S1]. For the fit the following parameters were used: a scale factor, surface displacement parameter of the first Co surface layer, displacement and occupation parameter for CO. A change of the occupation of the topmost CO layers did not improve the fit, indicative for an atomically smooth surface. The structure factors were extracted from the high energy XRD data following the procedure described in the literature[S2]. We first show the maximum projected images of the four conditions we have studied in Supplementary Figure 1. The images under the different conditions almost look identical, as the changes of the crystal surface are minute and reflect the findings that Co stays metallic and does not form surface reconstructions during the reaction or major deposits that make other structures appear (i.e. carbides) in line with previous studies[S3]. The used gas mixture is a total pressure of 1bar distributed between 1:2 mix of CO and H_2 of the specified partial pressure and the remainder being Ar. For example 200mbar indicates 1:2 CO:H_2 mixture of 200mbar with 800mbar of Ar. The flows were set to 10mln/min total applying flow ratios based on the described partial pressures relative to 1bar total pressure.

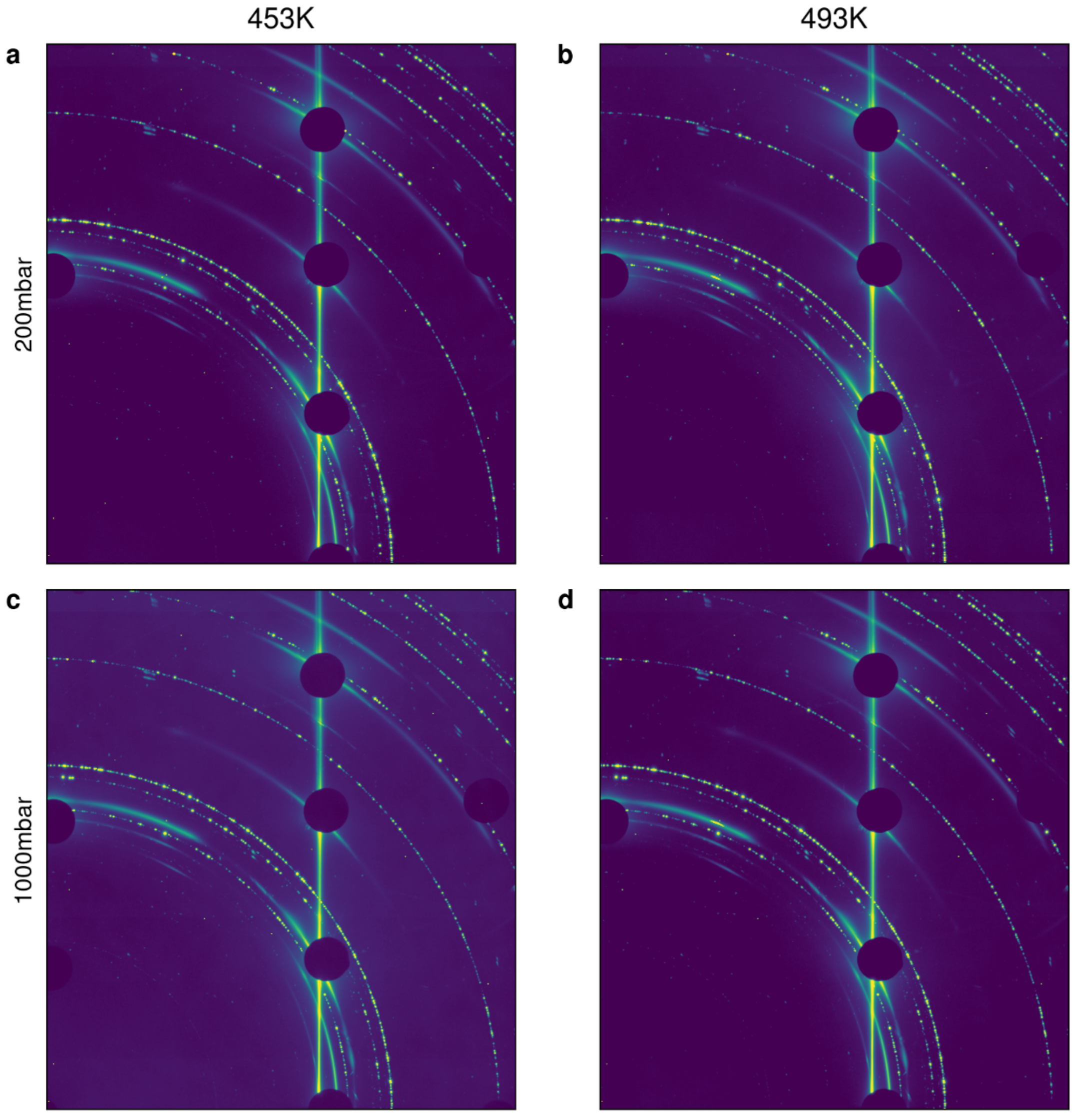

Supplementary Figure 1. **SXRD images of the Co(0001) surface under the indicated conditions in a 1:2 $CO{:}H_2$ reaction mixture.** Clear (1,0) rods are observed together with powder rings from surficial imperfections. All conditions resemble each other remarkably well, in line with the established observation that the Co substrate does not change under the reaction. Image is cropped to the top right quadrant of the detector, the direct beam behind beam stop is in the bottom left corner of. Presented in log-intensity.

In Supplementary Figure 2. we show the integrated structure factor from the four indicated conditions (200mbar and 1000mbar reactants at 456K and 496K). Comparing these lines, it is obvious that we observe no obvious differences. There seems to be an outlier at 1000mbar 456K around 2.5 r.l.u. (reciprocal lattice unit). The structure factor is higher around 0.5 r.l.u. for the 493K samples indicating an annealing like behavior under the reaction at the elevated temperatures. Peaks at ~0.7 and ~1.4 r.l.u. indicate a small and approximately constant amount

of fcc Co powder rings that are identifiable in the max projected images of Supplementary Figure 1. The fcc contribution can be attributed to the fact that the annealing temperature of the Co crystal is not too far away from the hcp->fcc transition temperature of 693K and thus a small fraction of the crystal surface might already undergo this transition. We estimate that significantly less than 1% of the crystal surface is reconstructed to the fcc-lattice based on the intensity of the fcc-related peaks and thus deem their influence on the reaction to be negligible.

The intensity associated with fcc at its maximum is 5000 intensity units. The shape of Bragg reflection suggest that they originate from small fcc domains as their Bragg spot is not very pronounced and they are rather smeared out. Their width is about 20x the width of the CTR of the hcp Co surface. Since we need to account for its width along its orthogonal, we assume it to be similar and thus add another factor of 20x leading to a total intensity of 2×10^6 intensity units. Our observed Bragg peaks of the hcp fraction in the fit lie in the regime of 10^8 to 10^9 which allows us to place an upper border of 1% fcc fraction and thus we deem its contribution small.

At 1000mbar and 503K the intensity of the rod decays slower which can be understood as a smoother surface.

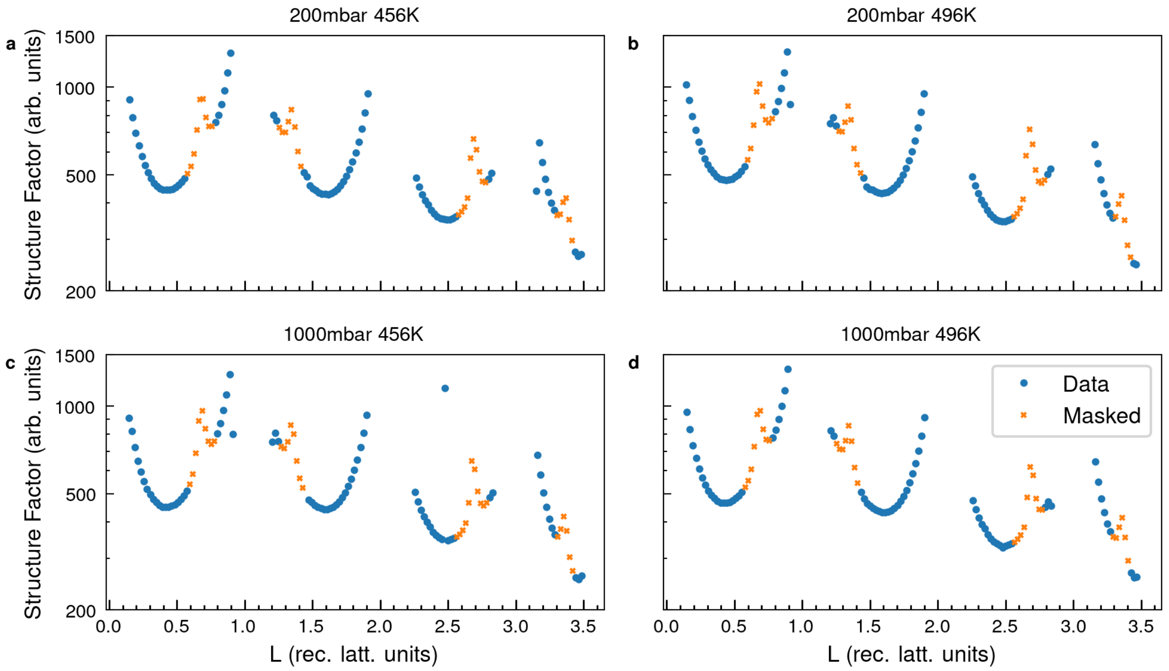

Supplementary Figure 2. **Integrated structure factor of the indicated conditions.** Around 1.0 and 2.0 rlu a beam stop was utilized, thus this region is ignored. The small FCC like Co peaks are plotted in yellow and are masked out for the fit analysis in the main manuscript.

S2 Compiled XPS Data

In S6.a and b we show the O *1s* and Co *2p* spectra that were omitted in the main text for the reader's convenience.

S2.a O *1s*

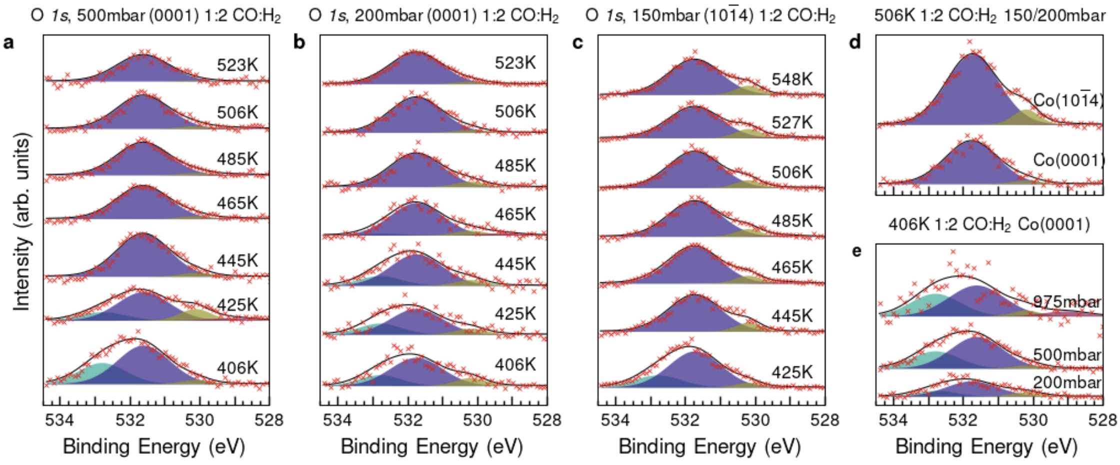

Supplementary Figure 3. **Compiled O 1s data for the indicated conditions.**

S2.b Co *2p*$_{3/2}$

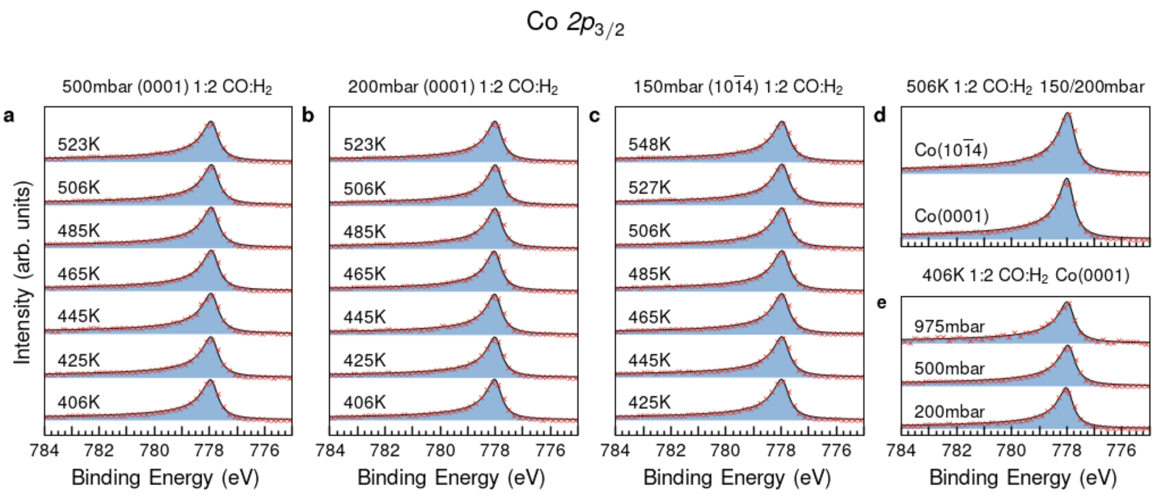

Supplementary Figure 4. **Compiled Co *2p*$_{3/2}$ data for the indicated conditions.**

S2.c Quantification of C and O Species

To quantify the surfacial coverages of C and O on the flat/stepped Co surfaces we utilize a bilayer model that we have described in our previous works[S4]. The inputs are the unit cell of the material[S5], photoionization cross-sections[S6], X-ray penetration depth[S7,8], photoelectron

inelastic mean free paths[S9], total scattering cross-section of the gas mixture[S10,11], incidence angle and detector geometry. In accordance with previous reports using this procedure we estimate a large error bar on the absolute value of these calculations of about 50% but very small (i.e. 5%) between spectra at different temperatures, pressures, and substrates[S4,12]. The repeatability of the measurements we estimate to be on the order of 10% and thus allowing us to make good comparisons, especially within an isobar. In our analysis we notice a strong pressure for the total signal, yet a minor variation for CO_{top}. Thus, we utilize the observed small T dependence of CO_{top} to calibrate the absolute coverages to the known value of 1/3 ML for this species by averaging the obtained coverages through our model for $T \in [425\text{K}, 506\text{K}]$. Compare Supplementary Figure 5 for an example depiction of this observation. This single number division ensures compatibility of average coverages but retains trends observed in the data. The C *1s* data in Fig. 2 of the main text is normalized in this way to enable a representative comparison of the surface species.

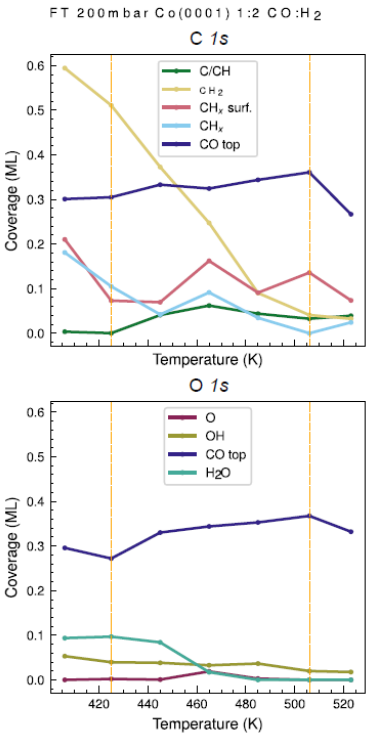

Supplementary Figure 5. **Coverages of the 200mbar experiments detailing the CO_{top} coverage as function of temperature for C *1s* (top) and O *1s* (bottom) spectra.** The trend that the coverage of this species is stable is found in all experiments and forms the basis for the recalibration by its average in the T region defined in the text. The vertical lines indicate the averaging T interval applied for the normalization.

S2.d Negligible influence of Recoil on the XPS Measurements

In our work we have carefully evaluated binding energy positions of known peaks and find that they do not exhibit a noticeable effect of the photoelectron recoil[S13]. The expected value for recoil energies should be $E_{recoil} \approx 200\text{meV}$ for C *1s* photo electrons emitted after excitation with a photon energy of 4600eV. This value is to be taken relative to the Fermi Energy shift of the substrate, Co which is much heavier indicating we should expect an even smaller shift from it, if any. We thus assume that the observed metal Fermi level energy position is so close to the undisturbed value that we do not observe it in our measurement. However, our observed peaks in CO_{top} fit the literature values very well[S14]. We explain this finding, that we are at temperatures where the surface layer's Debye temperature Θ_D is significantly larger than our

experimental temperature. This criterion is previously found to drastically increase the probability of a recoilless emission[S15]. The CO layer can be seen as a very stiff surface, comparable in compressibility to Pb on Ag(111)[S16]. The speed of sound is proportional to the compressibility and that in turn is directly proportional to the Debye temperature, which qualitatively shows that our assumption of the experiments being conducted significantly below Θ_D likely is true. Thus, we accept that recoil only plays a minor role in our analysis. This is further supported by observing that the energy width ($\approx$ 300meV) is larger than the recoil effect.

S2.e Mitigation of X-ray Induced Changes

To mitigate X-ray beam induced effects on our sample studies have been performed in each beam time where we find the cut-off where accumulation of beam induced species (i.e. graphite at 284.9-285.0eV), was observed. This criterion has been achieved by attenuating the full beam of P22 by a factor of 5x. As this species is not a peak in all our reported spectra the effectiveness of this method is shown. We perform this analysis directly after focusing the beam horizontally. Small beam pointing errors in the beamline can lead to a focus shift and consequently a larger beam. This acts like a further attenuation, as larger beams have a smaller fluence and to a reduction of beam damage and thus is not further considered problematic to the interpretation of the data. For the much higher energy work at P21 we used the full beam as it is expected that the exponentially smaller photoionization cross-sections at 10x higher energy will lead to negligible influences on the surface structure.

S2.f Gas-phase and Adsorbate Spectra

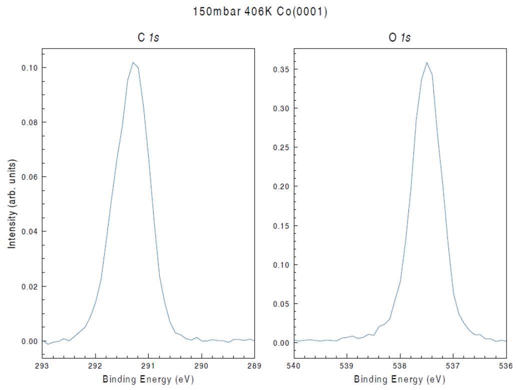

Supplementary Figure 6. **Gas phase spectrum of the C *1s* and O *1s* core-level for CO.**

The gas-phase and adsorbate XPS spectrum of carbon monoxide is depicted here in Supplementary Figure 6 under reaction conditions at the indicated pressure and temperature. This is representative of all gas-phase spectra in that we only observe a single peak without an indication of the reaction products. We attribute this to the fact that our reactor has a very short residence time of the gas (~1ms), due to the front cone design, while the reaction is expected to turn over at a rate of 100s per turnover. Thus, the products would be on a scale of 1 per hundred thousand which is lower than typical detection limits for XPS (parts per thousand levels).

S3 Mass Spectrometry

Mass-spectrometry is limited in its applicability as the flows are very high and the production rates which are very low. In other works we have shown that our sensitivity for the pressures studied here is on the order of TOF comparable to 10s of turnovers per site and second[S17,18]. The accepted rates on B5 rich surfaces of hydrogenation on Co are 300x lower ($3 \times 10^{-2} \frac{1}{\text{site}\cdot s}$) [S19]. Thus, operando quantification of the product distribution is all but impossible in the current setup for single crystal Co based Fischer-Tropsch synthesis.

S4 Computational Details

Density-Functional Theory (DFT) calculations were performed with the GPAW (generalized plane augmented wave)[S20,21] software using PAW:s. Calculations were done using a slab with four layers of metal atoms where the molecules are adsorbed at the surface. Since Co is magnetic, we have used spin-polarization in all calculations. The resulting magnetic moment observable was $1.8 \frac{\mu_B}{\text{atom}}$ well in line with literature[S22]. The system is built so the equivalent of ¼ ML coverage is produced as shown in Supplementary Figure 7. The bottom two metal layers of the slab are frozen with respect with bulk Co while the other degrees of freedom are optimized, so we have a proper relaxation of the molecule adsorbed in different sites and all the metallic atoms surrounding it.

The finite-difference mode with grid spacing of 0.2 Å was used in conjunction with a Monkhorst–Pack k-point sampling density of 2.5 k-points/Å^3 in the supercell and the revised Perdew-Burke-Ernzerhof (RPBE) exchange-correlation functional[S23]. For binding energy (BE) calculations we must make sure the slab's supercell is big enough (in our experience larger than 8 Å on each dimension) so core-hole interactions with its periodic images are reduced. The k-point sampling density was also increased to at least 3.5 k-points/Å^3.

The absolute XPS BEs are calculated using generated core-ionized PAW:s (explicit core-hole) representing the final or excited state and the BE is obtained from the equation

$$BE = E_{\text{final}} - E_{gs}$$

where E_{final} is the total electronic energy of the final or excited state, and E_{gs} is the total electronic energy of the initial or ground state. The BEs are then shifted according to the binding energy position of CO_{top}.

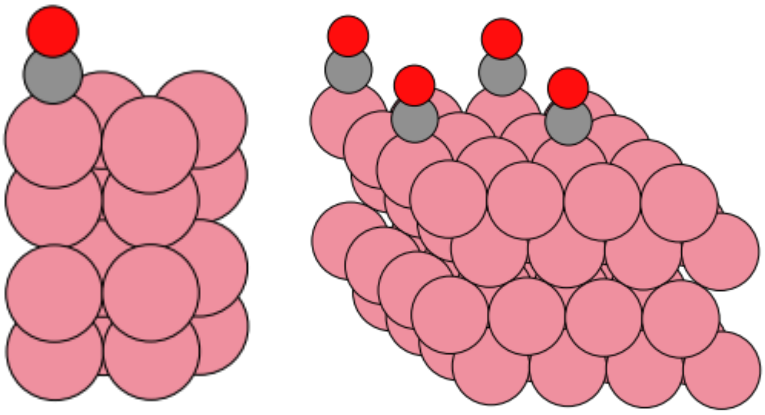

Supplementary Figure 7. **Example of periodic slabs for DFT calculations for CO adsorbed on-top of a Co(0001) surface.** On the left we have a more compact supercell that can be used for optimization purpose. On the right the supercell is doubled so we avoid core-hole interactions with its periodic images on BE calculations.

S5 Pressure Calibration

The pressure in the reaction zone is calibrated according to established procedures[S24]. In lieu of a micro pressure sensor that would be able to directly probe the conditions in the ~30µm sized virtual cell, we instead calibrate the pressure observed in the first differential pumping stage with the 1:2 CO:H_2 gas mixture and pressures in the chamber. An example graph of this pressure calibration is given in Supplementary Figure 8. The error of this measurement is estimated to be within the reproducibility of the two gauges involved (Pfeiffer RPT 200 PB, full scale accuracy $\pm 0.3\%$) resulting in a net uncertainty of $\pm 0.42\%$ which amounts to about 4 mbar at 1000mbar. Due to the particular style of recording used close to the end of the region 20mbar steps are taken, which further limits the uncertainty of the absolute pressure.

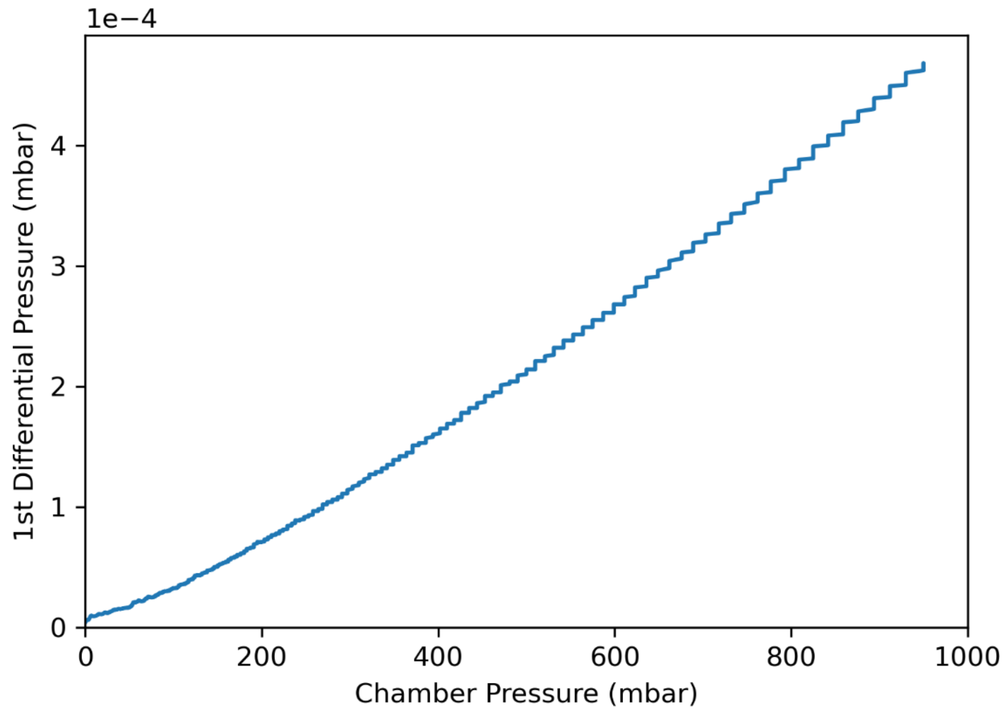

Supplementary Figure 8. Pressure calibration for 1:2 $CO:H_2$ mixture comparing the chamber pressure and the pressure of the first differential pumping stage.

S6. B5 Sites of the Co($10\bar{1}4$) Surface

In this particular surface cut the planes expose differently large terraces that can be 1-2 atoms long and exhibit symmetries of alternating planes consisting of 3-fold coordinated Co atoms and 4-fold coordinated Co atoms. Each adjacent site of a 4-fold plane that is below a 3-fold plane exposes a B5 type site where an adsorbed atom would be in the proximity of 5 surrounding neighbors (4 from the same plane and 1 from the 3-fold plane above this plane). This particular geometry facilitates an enhanced catalytic activity of the catalyst[S25] and is not existing in the flat Co(0001) surface, as its maximum coordination number is 4. A view onto this surface is presented in Supplementary Figure 9.

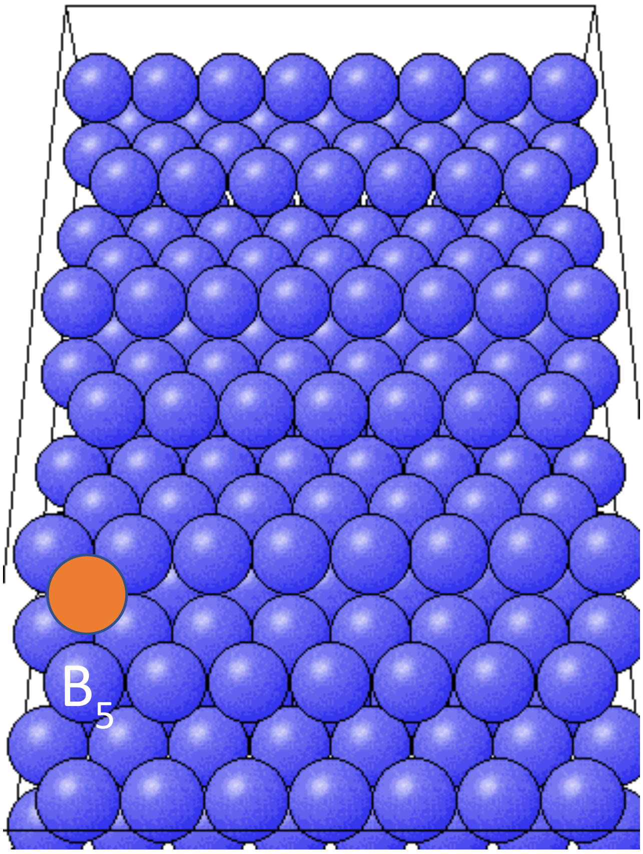

Supplementary Figure 9. **B5 Sites on Co($10\bar{1}4$).** Co($10\bar{1}4$)surface displayed from above, visualized with SurfaceExplorer. The intersection of the lower 4-fold coordinated terrace with the above-lying 3- fold coordinated terrace creates a unique 5-fold coordinated site that is denoted as B5 site and is connected to higher activity of this Co surface.

S7. Schematic Depiction of Possible Adsorbate Configuration

In this section we include Supplementary Figure 10, a graphical schematic depiction of the probable adsorbates in the colours as they are presented to the peaks in the main text for easy recognition for the C *1s* core level. In Supplementary Figure 11 we present the same but for the O *1s* core-level.

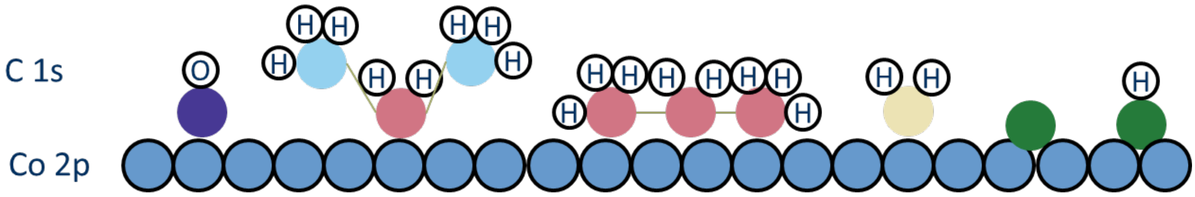

Supplementary Figure 10. **Schematic depiction of probable adsorbates for the C *1s* core-level.**

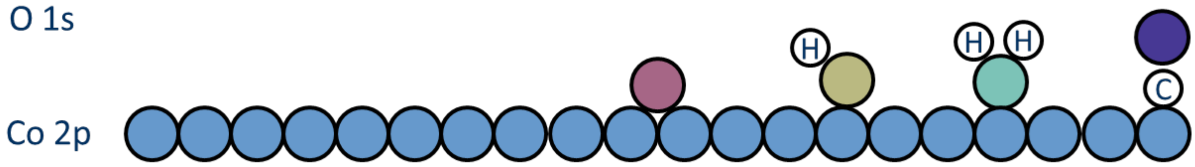

Supplementary Figure 11. **Schematic depiction of probable adsorbates for the O *1s* core-level.**

S8. Schematic Depiction of a Monolayer

In this work we define a monolayer as atoms occupying a site directly on top of all Co atoms making a full hcp layer. We are depicting the side view of this in Supplementary Figure 12.

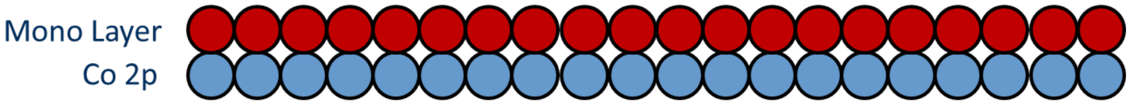

Supplementary Figure 12. **Schematic depiction of a monolayer as used in this work.**

References

S1. Vlieg, E. *ROD* : a program for surface X-ray crystallography. *J. Appl. Crystallogr.* **33**, 401–405 (2000).

S2. Gustafson, J. *et al.* High-Energy Surface X-ray Diffraction for Fast Surface Structure Determination. *Science* **343**, 758–761 (2014).

S3. Böller, B., Durner, K. M. & Wintterlin, J. The active sites of a working Fischer–Tropsch catalyst revealed by operando scanning tunnelling microscopy. *Nat. Catal.* **2**, 1027 (2019).

S4. Degerman, D. *et al.* State of the Surface During CO Hydrogenation over Ni(111) and Ni(211) Probed by Operando X-ray Photoelectron Spectroscopy. *J. Phys. Chem. C* acs.jpcc.2c07650 (2023) doi:10.1021/acs.jpcc.2c07650.

S5. None Available. Materials Data on Co by Materials Project. (2020) doi:10.17188/1263614.

S6. Trzhaskovskaya, M. B. & Yarzhemsky, V. G. Dirac–Fock photoionization parameters for HAXPES applications. *At. Data Nucl. Data Tables* **119**, 99–174 (2018).

S7. Parratt, L. G. Surface studies of solids by total reflection of x-rays. *Phys. Rev.* **95**, 359–369 (1954).

S8. Henke, B. L., Gullikson, E. M. & Davis, J. C. X-Ray Interactions: Photoabsorption, Scattering, Transmission, and Reflection at E = 50-30,000 eV, Z = 1-92. *At. Data Nucl. Data Tables* **54**, 181–342 (1993).

S9. Shinotsuka, H., Tanuma, S., Powell, C. J. & Penn, D. R. Calculations of electron inelastic mean free paths. X. Data for 41 elemental solids over the 50 eV to 200 keV range with the relativistic full Penn algorithm: Calculations of electron inelastic mean free paths. X. *Surf. Interface Anal.* **47**, 871–888 (2015).

S10. Danilatos, G. D. Optimum beam transfer in the environmental scanning electron microscope. *J. Microsc.* **234**, 26–37 (2009).

S11. García, G., Roteta, M. & Manero, F. Electron scattering by N2 and CO at intermediate energies: 1–10 keV. *Chem. Phys. Lett.* **264**, 589–595 (1997).

S12. Shipilin, M. *et al.* In Situ Surface-Sensitive Investigation of Multiple Carbon Phases on Fe(110) in the Fischer–Tropsch Synthesis. *ACS Catal.* **12**, 7609–7621 (2022).

S13. Takata, Y. *et al.* Recoil effects of photoelectrons in a solid. *Phys. Rev. B* **75**, 233404 (2007).

S14. Weststrate, C. J. (Kees-J. *et al.* Mechanistic insight into carbon-carbon bond formation on cobalt under simulated Fischer-Tropsch synthesis conditions. *Nat. Commun.* **11**, 750 (2020).

S15. Suga, S. *et al.* Do all nuclei recoil on photoemission in compounds? *New J. Phys.* **11**, 073025 (2009).

S16. Quiros, C., Robach, O., Isern, H., Ordejón, P. & Ferrer, S. Compressibility of CO adsorbed on Ni from 10−6 mbar to 1.2 bar ambient CO pressures investigated with X-ray diffraction. *Surf. Sci.* **522**, 161–166 (2003).

S17. Degerman, D. *et al.* Operando Observation of Oxygenated Intermediates during CO Hydrogenation on Rh Single Crystals. *J. Am. Chem. Soc.* **144**, 7038–7042 (2022).

S18. Amann, P. *et al.* The state of zinc in methanol synthesis over a Zn/ZnO/Cu(211) model catalyst. *Science* **376**, 603–608 (2022).

S19. Weststrate, C. J., van Helden, P. & Niemantsverdriet, J. W. Reflections on the Fischer-Tropsch synthesis: Mechanistic issues from a surface science perspective. *Catal. Today* **275**, 100–110 (2016).

S20. Enkovaara, J. *et al.* Electronic structure calculations with GPAW: a real-space implementation of the projector augmented-wave method. *J. Phys. Condens. Matter* **22**, 253202 (2010).

S21. Mortensen, J. J., Hansen, L. B. & Jacobsen, K. W. Real-space grid implementation of the projector augmented wave method. *Phys. Rev. B* **71**, 035109 (2005).

S22. Murthy, V. S. R. *Structure And Properties Of Engineering Materials*. (McGraw-Hill Education (India) Pvt Limited, 2003).

S23. Hammer, B., Hansen, L. B. & Nørskov, J. K. Improved adsorption energetics within density-functional theory using revised Perdew-Burke-Ernzerhof functionals. *Phys. Rev. B* **59**, 7413–7421 (1999).

S24. Amann, P. *et al.* A high-pressure x-ray photoelectron spectroscopy instrument for studies of industrially relevant catalytic reactions at pressures of several bars. *Rev. Sci. Instrum.* **90**, 103102 (2019).

S25. ten Have, I. C. & Weckhuysen, B. M. The active phase in cobalt-based Fischer-Tropsch synthesis. *Chem Catal.* **1**, 339–363 (2021).
