# Supplementary figures and images for: In-situ probing of the Fischer-Tropsch reaction on Co single crystal surfaces up to 1 bar
Source: Nat Commun. 2025 Jan 24;16:1005. doi: 10.1038/s41467-025-56082-8 (PMC11761050; doi:10.1038/s41467-025-56082-8)

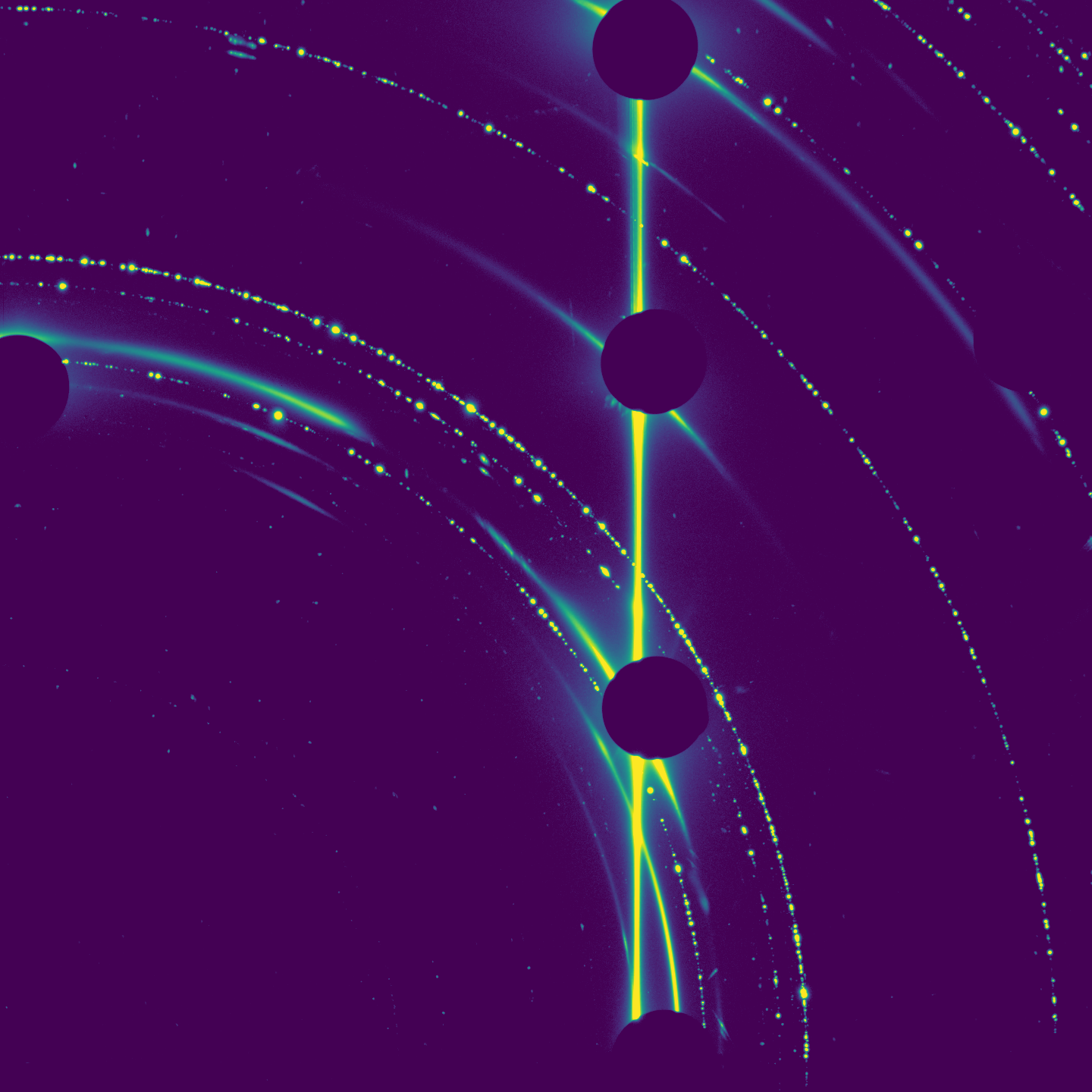

Supplement: Supplementary file 3 — Source Data [file 41467_2025_56082_MOESM3_ESM.zip › Fig1/sxrd g.png]
